# Supplementary material for: SCAPP: an algorithm for improved plasmid assembly in metagenomes
Source: Microbiome. 2021 Jun 25;9:144. doi: 10.1186/s40168-021-01068-z (PMC8228940; doi:10.1186/s40168-021-01068-z)
Supplement: Supplementary file 2 — Supplementary methods, experimental settings information, and results supporting the main text of this paper, including Figures S1-S4, Supplementary Tables 1 and 2. PDF file. [file 40168_2021_1068_MOESM2_ESM.pdf]

Supplementary information for SCAPP: An algorithm for improved plasmid assembly in metagenomes

David Pellow, Alvah Zorea, Maraike Probst, Ori Furman, Arik Segal, Itzhak Mizrahi, and Ron Shamir

January 10, 2021

S1 Alternatives for user set parameters

The SCAPP pipeline is highly flexible, and many of the options and parameters can be set by the user. In most cases, we recommend using the default options and settings. Some of the alternatives that can be chosen by the user are described below. All of the parameter settings that may be changed by the user are fully documented at: `https://github.com/Shamir-Lab/SCAPP`.

Read mapping: The user has the option of providing a sorted and indexed BAM alignment file created by any method.

Plasmid-specific genes: The user may add any set of PSGs or remove any of those included with SCAPP.

Plasmid classification scores: The sequences may be classified using PlasFlow and the PlasFlow classification output file can be provided to SCAPP.

Algorithm thresholds: Thresholds for finding plasmid gene matches, defining probable plasmid and chromosomal sequences, identifying potential plasmids, filtering them, and many more can all be user-defined. The full software documentation at `https://github.com/Shamir-Lab/SCAPP` details all of these user options.

S2 Plasmid-specific genes

We created four sets of plasmid-specific genes (PSGs) by database mining and expert curation:

1. MOB genes: 890 amino acid sequences of plasmid maintenance genes curated by plasmid biologists from the Mizrahi Lab (Ben-Gurion University) and filtered computationally (see details of filtering below).
2. Plasmid ORFs: 4276 nucleotide sequences corresponding to ORFs annotated with 'mobilization', 'conjugation', 'partitioning', 'toxin-antitoxin', 'replication', or 'recombination' from a large set of putative plasmids found by the Mizrahi Lab and then filtered computationally.
3. ACLAME plasmid genes: 4813 nucleotide sequences of genes that make up 96 gene families in the ACLAME database [1] that were manually selected as possibly plasmid-specific. The set of genes was deduplicated and filtered computationally.
4. PLSDB-specific ORFs: 94478 plasmid-specific sequences determined as follows: We used MetaGeneMark [2] to predict genes in the plasmid sequences from PLSDB (v.2018_12_05) [3]. We then counted the number of BLAST matches ($> 75\%$ identity match along $> 75\%$ of the gene length) to these genes in both PLSDB and bacterial reference genomes from NCBI (downloaded January 9, 2019). We

considered each predicted gene that appeared in the plasmids more than 20 times and was $> 20\times$ more prevalent in the plasmids than in the genomes to be plasmid-specific.

Sets 1–3 were filtered as follows: We counted matches between the sequences and PLSDB plasmids and NCBI bacterial reference genomes as for the PLSDB-specific ORFs (set 4). We excluded any gene that had more than 4 matches to bacterial genes *and* met one of the following conditions: (1) ≤ 4 matches to plasmid genes and $> 4\times$ as many matches to bacterial genes as plasmid genes; or, (2) > 4 plasmid gene matches, but $\leq 4\times$ as many matches to plasmid genes as to bacterial genes.

We did not search for and remove duplicate genes between sets, and did not back-translate the amino acid sequences.

S3 Potential plasmid cycle criteria

Once the set of lightest cycles has been generated, each cycle is evaluated as a potential plasmid based on its structure in the assembly graph, the PSGs it contains, its plasmid score, paired-end read links, and coverage uniformity. A cycle is defined as a potential plasmid if one of the following criteria is met:

1. The cycle is formed by an isolated "compatible" self-loop node v, i.e. $len(v) > 1000$, $indeg(v) = outdeg(v) = 1$, and at least one of the following conditions holds:
   (a) v has a high plasmid score $s(v) > 0.9$.
   (b) v has a PSG hit.
   (c) $< 10\%$ of the paired-end reads with a mate on v have the other mate on a different node.
2. The cycle is formed by a connected compatible self-loop node v, i.e. $len(v) > 1000$, $indeg(v) > 1$ or $outdeg(v) > 1$, and $< 10\%$ of the paired-end reads with a mate on v have the other mate on a different node.
3. The cycle is not formed by a self-loop and has:
   (a) Uniform coverage: $CV(C) < 0.5$, and
   (b) Consistent mate-pair links: a node in the cycle is defined as an "off-path dominated" node if the majority of the paired-end reads with one mate on the node have the other mate on a node that is not in the cycle. If less than half the nodes in the cycle are "off-path dominated", then we consider the mate-pair links to be consistent.

S4 Simulation of metagenomes with plasmids

To create the simulated metagenomes, we downloaded all completed whole genome bacterial reference sequences from RefSeq (RefSeq database updated on March 11, 2020). We first compiled a list of bacterial strains or species that have been previously identified as prevalent in the human gut from three sources: (1) The list compiled by Alneberg *et al.* [4] (Sup Table 1). (2) Species with abundance > 0.01 in at least one human gut sample from the human microbiome project (HMP1) [5] as estimated by MetaPhLan (abundance table available from `https://www.hmpdacc.org/HMSMCP/#data`). (3) The "dominant species" identified by Forster *et al.* [6] (Sup Table 5) in the HGG (Human Gastrointestinal Bacteria Genome Collection). We searched for the strains or species on this combined list in the RefSeq database, giving preference to strain level matches. When multiple references appeared (for example, when a listed species has multiple reference strains), we gave preference to those with longer plasmids (>10kbp), followed by those with any plasmid, choosing randomly between references with the same preference. The list of human gut specific bacteria used in the simulations contained 145 references, and is provided in Additional file 2.

For each simulation we first selected from the human gut specific bacteria and then supplemented with randomly selected reference sequences to reach the desired number of genomes. Since the plasmids sequenced with completed whole bacterial genomes are usually long, we also supplemented with a fixed number of shorter

(<10kbp) plasmids, selected randomly and associated at random with host genomes in the simulation. (5 short plasmids were added in Sim1, 15 in Sim2, 50 in Sim3 and Sim4, 100 in Sim5, 150 in Sim6, and 200 in Sim7.)

Genome abundance and plasmid copy number were assigned using realistic distributions. For genome abundance we used the log-normal distribution ($\mu = 1.5$, $\sigma = 1$), normalized so that the relative abundances sum to 1. This long-tailed distribution mimics the abundance distribution of real microbiome samples. Plasmids were assigned the same abundance as their hosts, and plasmid copy number was assigned according to one of several geometric distributions according to the plasmid length. The parameter of the geometric distribution of a plasmid of length L was set to be

$$p = \begin{cases} log_{10}(L)/30, & 1\text{kbp} \leq L < 10\text{kbp} \\ log_{10}(L)/20, & 10\text{kbp} \leq L < 100\text{kbp} \\ log_{10}(L)/10, & 100\text{kbp} \leq L < 1\text{Mbp} \\ 1, & L \geq 1\text{Mbp} \end{cases}$$

This makes it more likely for shorter plasmids to have higher copy numbers, in accordance with observed plasmid copy number patterns.

Paired-end short reads were simulated from the genome references using InSilicoSeq [7] with the HiSeq error model (default read length = 126bp). To reflect circularity of the plasmids and bacterial genomes, multiple copies of the reference sequence were concatenated before generating reads.

S5 Experimental settings and evaluation

All metagenomes were assembled using the SPAdes assembler (v3.13) with the `--meta` option. The default of 16 threads were used, and the maximum memory was set to 750 GB. metaplasmidSPAdes (mpSpades) was run with the same parameters. mpSpades internally chooses the maximal value of k to use for the k-mer length in the assembly graph. We matched the values of k used in SPAdes to these values for each dataset. Defaults were used for all other options for Recycler and SCAPP. In practice, the maximum k value was 77 for the simulations and human metagenomic samples, and 127 for the plasmidome and parallel metagenome-plasmidome samples.

For a simulated metagenome, the set of reference plasmids included in the simulation that were covered along $> 95\%$ of their length by simulated reads was used as the gold standard. Reads were mapped using BWA [8], and coverage at each base of the reference plasmids was called using bedtools [9].

We used BLAST to match the assembled plasmids to the gold standard plasmid sequences. A plasmid assembled by one of the tools was considered to be a true positive if $> 90\%$ of its length was covered by BLAST matches to $> 90\%$ of a reference with $> 80\%$ sequence identity. The rest of the assembled plasmids were considered to be false positives. Gold standard plasmids that did not have assembled plasmids matching them were considered to be false negatives. Precision was defined as $TP/(TP + FP)$ and recall was defined as $TP/(TP + FN)$, where TP, FP, and FN were the number of true positive, false positive, and false negative plasmids, respectively. The F1 score was defined as the harmonic mean of precision and recall. Precision, recall, and F1 values are adjusted to percentage throughout. Note that these metrics evaluate the complete assembly of entire plasmids, and do not capture rates of local misassembly or short range assembly errors within each assembled plasmid.

For the human microbiome and plasmidome samples, the set of plasmids serving as the gold standard was selected from PLSDB (v.2018_12_05) [3], a large curated plasmid database. After filtering duplicate plasmids, the PLSDB contains 13469 reference plasmids. The contigs from the metaSPAdes assembly were matched against the plasmids in PLSDB using BLAST. Matches between a contig and a reference plasmid with sequence identity $> 85\%$ were marked and a contig was said to match a reference if $> 85\%$ of its length was marked. Reference plasmids with $> 90\%$ of their lengths covered by marked regions of the matching contigs were used as the gold standard.

Table 1 Full performance on simulated metagenome datasets. The gold standard is the number of plasmids in the simulation that are covered by simulated reads (# covered).

		Recycler			mpSpades			SCAPP		
Sample	# covered	precision	recall	F1	precision	recall	F1	precision	recall	F1
Sim1	9	57.1	44.4	50.0	100	11.1	20.0	80.0	44.4	57.1
Sim2	37	60	32.4	40.1	100	24.3	39.1	56.5	35.1	43.3
Sim3	136	52.5	23.9	32.8	96.3	19.4	32.3	81.3	29.1	42.9
Sim4	132	62.9	30.2	40.8	100	22.3	36.5	86.3	34.1	48.9
Sim5	253	55.6	25.7	35.2	96.2	20.5	33.8	77.2	34.3	47.5
Sim6	368	51.4	19.7	28.5	96.6	15.8	27.1	72.9	24.4	36.5
Sim7	410	62.9	20.6	31.1	95.7	16.5	28.1	75.9	27.6	40.5

The set of plasmids assembled by each method was compared to the gold standard set using BLAST. A predicted plasmid was considered a true positive if there were sequence matches at $> 80\%$ identity between the plasmid and a gold standard plasmid that covered more than 90% of their lengths.

Note that in the case of the real samples, if two assembled plasmids matched to the same reference gold standard plasmid sequence(s), then one of them was considered to be a false positive. This strict definition penalized methods for unnecessarily splitting potential plasmid genomes into multiple different plasmids. If there were multiple gold standard reference plasmids that were matched to a single assembled plasmid, then none of them was considered as a false negative. The precision, recall, and F1 score were calculated as for the simulation.

For the parallel metagenome-plasmidome sample, plasmidomic reads were aligned to the plasmid sequences and metagenome assembly contigs using BWA [8]. Coverage at each base of each metagenomic contig was called using bedtools [9].

To compare the overlap between plasmids identified by the different tools, we considered two plasmids to be the same if their sequences matched at $> 80\%$ identity across $> 90\%$ of their length. For visualization purposes, when two plasmids in one tool match one plasmid in another, they are represented as one overlap in the venn diagram (Figures S1 and S3).

S6 Extended results for simulated datasets

Table 1 reports the full precision, recall, and F1 performance results for all tools on the simulated metagenome datasets. Table 2 reports the performance results when split by length into shorter (< 10 kbp) and longer (≥ 10 kbp) plasmids. Figure S1 shows the overlap between the plasmids assembled by each tool in the simulated metagenomes.

Table 2 Performance on simulated metagenome datasets stratified by short plasmids (< 10 kbp) and long plasmids (≥ 10 kbp). The number of gold-standard plasmids (GS) for each length bin is indicated in parentheses.

		Recycler				mpSpades				SCAPP			
Sample	Length bin (# GS)	# plasmids	precision	recall	F1	# plasmids	precision	recall	F1	# plasmids	precision	recall	F1
Sim1	< 10 kb (5)	5	80.0	80.0	80.0	1	100.0	20.0	33.3	4	100.0	80.0	88.9
	≥ 10 kb (4)	2	0.0	0.0	0.0	0	-	0.0	0.0	1	0.0	0.0	0.0
Sim2	< 10 kb (18)	18	66.7	66.7	66.7	7	100.0	38.9	56.0	17	70.6	66.7	68.6
	≥ 10 kb (19)	2	50.0	5.3	9.5	2	50.0	5.3	9.5	6	33.3	10.5	16.0
Sim3	< 10 kb (69)	55	50.9	41.8	45.9	22	95.5	31.3	47.2	37	89.2	49.3	63.5
	≥ 10 kb (67)	6	66.7	8.0	11.0	5	100.0	7.5	13.9	11	54.5	9.0	15.4
Sim4	< 10 kb (63)	57	61.4	58.3	59.8	25	100.0	41.0	58.1	42	90.5	62.3	73.8
	≥ 10 kb (69)	5	80.0	5.8	10.8	4	100.0	5.8	11.0	9	66.7	8.8	15.6
Sim5	< 10 kb (128)	106	56.6	48.4	52.2	48	95.8	37.1	53.5	80	90.0	58.5	70.9
	≥ 10 kb (125)	9	44.4	3.2	6.0	5	100.0	4.0	7.7	30	43.3	10.4	16.8
Sim6	< 10 kb (196)	125	52.0	34.6	41.5	51	96.1	25.8	40.7	88	87.5	42.3	57.0
	≥ 10 kb (172)	13	46.2	3.5	6.5	8	100.0	4.7	8.9	30	33.3	5.8	10.0
Sim7	< 10 kb (225)	121	61.2	34.1	43.8	61	95.1	26.9	41.9	109	86.2	46.5	60.5
	≥ 10 kb (185)	11	81.8	4.9	9.2	8	87.5	3.8	7.3	32	40.6	7.1	12.0

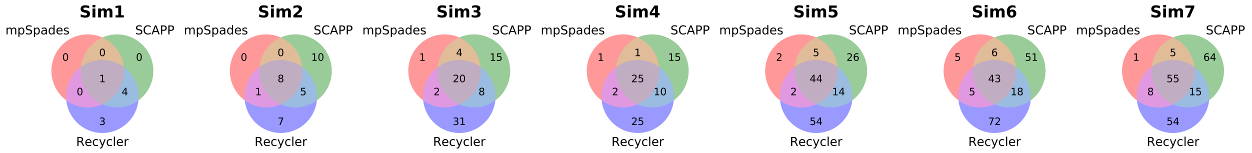

Figure S1 Plasmid overlap between tools in simulation. Overlap of the plasmids assembled by the tools on each of the simulated metagenomes.

S7 Extended results for human metagenomes

Figure S2 presents the F1 scores of the plasmid assemblers across all human gut metagenome samples. Table 3 reports the full results and the number of plasmids assembled by each tool and the median plasmid length for each of the human gut microbiome samples.

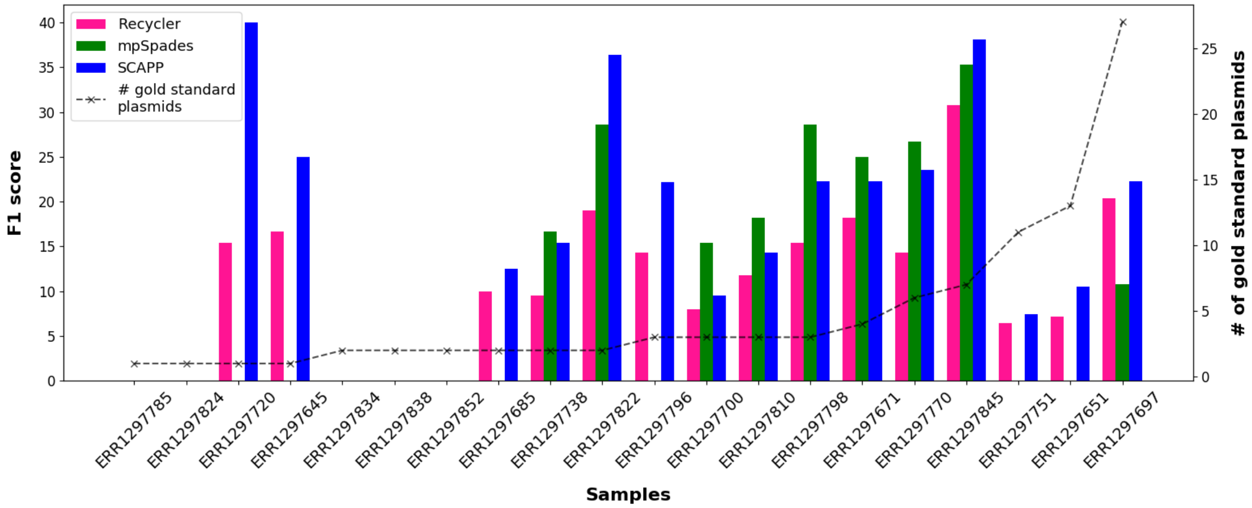

Figure S2 Results on 20 human gut metagenomes. F1 scores of the plasmids assembled by Recycler, mpSpades and SCAPP in the human gut microbiome samples (accessions given on x-axis), calculated using PLSDB plasmids as the gold standard. The dashed line shows the number of gold standard plasmids in each sample. Where bars are omitted the F1 score was 0.

Table 3 Full results on the human gut microbiome samples and number of plasmids and median lengths (in kbp).

	Gold standard	Recycler				mpSpades				SCAPP			
Sample	# plasmids (median length)	# plasmids (median length)	Precision	Recall	F1	# plasmids (median length)	Precision	Recall	F1	# plasmids (median length)	Precision	Recall	F1
ERR1297785	1 (6.0)	14 (2.4)	0	0	0	4 (4.7)	0	0	0	8 (4.3)	0	0	0
ERR1297824	1 (6.0)	15 (3.2)	0	0	0	6 (5.2)	0	0	0	8 (4.8)	0	0	0
ERR1297720	1 (2.7)	12 (3.4)	8.3	100.0	15.4	3 (4.2)	0	0	0	4 (3.4)	25.0	100.0	40.0
ERR1297645	1 (2.7)	11 (3.2)	9.1	100.0	16.7	7 (5.2)	0	0	0	7 (4.5)	14.3	100.00	25.0
ERR1297834	2 (4.1)	5 (4.4)	0	0	0	3 (6.4)	0	0	0	3 (6.3)	0	0	0
ERR1297838	2 (6.5)	17 (2.0)	0	0	0	4 (4.6)	0	0	0	8 (5.4)	0	0	0
ERR1297852	2 (19.5)	17 (5.1)	0	0	0	5 (5.3)	0	0	0	6 (5.2)	0	0	0
ERR1297685	2 (2.5)	18 (5.2)	5.6	50.0	10.0	7 (6.1)	0	0	0	14 (4.3)	7.1	50.0	12.5
ERR1297738	2 (19.5)	19 (5.1)	5.3	50.0	9.5	10 (4.9)	10.0	50.0	16.7	11 (5.1)	9.1	50.0	15.4
ERR1297822	2 (2.2)	19 (4.4)	10.5	100.0	19.0	5 (4.5)	20.0	50.0	28.6	9 (4.1)	22.2	100.0	36.4
ERR1297796	3 (8.9)	11 (2.9)	9.1	33.3	14.3	5 (3.6)	0	0	0	6 (2.8)	16.7	33.3	22.2
ERR1297700	3 (5.6)	22 (2.9)	4.5	33.3	8.0	10 (4.4)	10.0	33.3	15.4	18 (4.4)	5.6	33.3	9.5
ERR1297810	3 (5.6)	14 (3.5)	7.1	33.3	11.8	8 (4.4)	12.5	33.3	18.2	11 (4.6)	9.1	33.3	14.3
ERR1297798	3 (8.9)	11 (2.9)	9.1	50.0	15.4	5 (6.4)	20.0	50.0	28.6	7 (2.9)	14.3	50.0	22.2
ERR1297671	4 (7.4)	8 (3.8)	12.5	33.3	18.2	5 (4.2)	20.0	33.3	25.0	6 (4.0)	16.7	33.3	22.2
ERR1297770	6 (8.9)	23 (3.4)	8.7	40.0	14.3	10 (4.7)	20.0	40.0	26.7	12 (4.4)	16.7	40.0	23.5
ERR1297845	7 (8.9)	20 (3.3)	20.0	66.7	30.8	11 (5.9)	27.3	50.0	35.3	15 (3.8)	26.7	66.7	38.1
ERR1297751	11 (4.9)	20 (4.0)	5.0	9.1	6.5	6 (5.6)	0	0	0	16 (4.4)	6.3	9.1	7.4
ERR1297651	13 (114.2)	15 (3.2)	6.7	7.7	7.1	4 (4.9)	0	0	0	6 (4.5)	16.7	7.7	10.5
ERR1297697	27 (2.1)	25 (3.8)	20.0	20.8	20.4	11 (5.4)	18.2	7.7	10.8	21 (4.5)	23.8	20.8	22.2

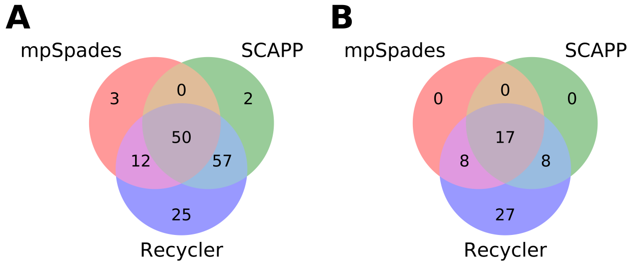

Figure S3 Number of plasmids assembled by each tool on the parallel samples. A: Plasmidome sample. B: Metagenome sample. Discrepancies between the numbers in the diagram and Table 4 are due to cases of overlaps between two plasmids in one tool to one plasmid in another, which were counted as one.

S8 Extended results for parallel plasmidome-metagenome

Figure S3 shows the overlap between the plasmids assembled by the tools in the parallel cow rumen plasmidome and metagenome samples.

Figure S4 shows the annotations of the gene functions and hosts for the plasmids assembled in the rumen plasmidome.

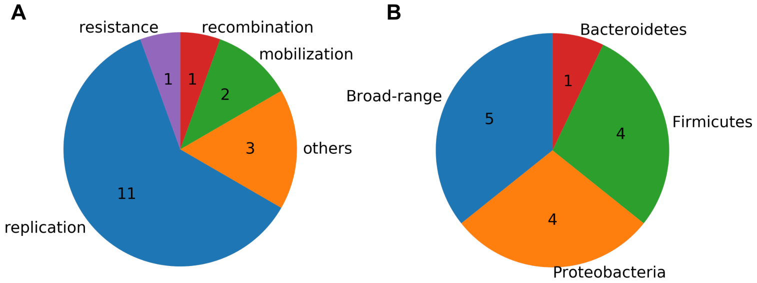

Figure S4 Annotation of genes on the plasmids identified by SCAPP in the rumen plasmidome sample. A: Functional annotations of the plasmid genes. B: Host annotations of the plasmid genes.

References

[1] Leplae, R., Lima-Mendez, G., Toussaint, A.: ACLAME: a classification of mobile genetic elements, update 2010. Nucleic Acids Research **38**(suppl_1), 57–61 (2009)

[2] Zhu, W., Lomsadze, A., Borodovsky, M.: Ab initio gene identification in metagenomic sequences. Nucleic Acids Research **38**(12), 132–132 (2010)

[3] Galata, V., Fehlmann, T., Backes, C., Keller, A.: PLSDB: a resource of complete bacterial plasmids. Nucleic Acids Research **47**(D1), 195–202 (2018)

[4] Alneberg, J., Bjarnason, B.S., De Bruijn, I., Schirmer, M., Quick, J., Ijaz, U.Z., Lahti, L., Loman, N.J., Andersson, A.F., Quince, C.: Binning metagenomic contigs by coverage and composition. Nature methods **11**(11), 1144–1146 (2014)

[5] Methé, B.A., Nelson, K.E., Pop, M., Creasy, H.H., Giglio, M.G., Huttenhower, C., Gevers, D., Petrosino, J.F., Abubucker, S., Badger, J.H., *et al.*: A framework for human microbiome research. nature **486**(7402), 215 (2012)

[6] Forster, S.C., Kumar, N., Anonye, B.O., Almeida, A., Viciani, E., Stares, M.D., Dunn, M., Mkandawire, T.T., Zhu, A., Shao, Y., *et al.*: A human gut bacterial genome and culture collection for improved metagenomic analyses. Nature biotechnology **37**(2), 186–192 (2019)

[7] Gourlé, H., Karlsson-Lindsjö, O., Hayer, J., Bongcam-Rudloff, E.: Simulating illumina metagenomic data with insilicoseq. Bioinformatics **35**(3), 521–522 (2018)

[8] Li, H.: Aligning sequence reads, clone sequences and assembly contigs with bwa-mem. arXiv preprint arXiv:1303.3997 (2013)

[9] Quinlan, A.R., Hall, I.M.: Bedtools: a flexible suite of utilities for comparing genomic features. Bioinformatics **26**(6), 841–842 (2010)
